# Supercongruences involving Domb numbers and binary quadratic forms

**DOI:** 10.1007/s13398-023-01509-4

**Published:** 2023-10-03

**Authors:** Guo-Shuai Mao, Michael J. Schlosser

**Affiliations:** 1 Department of Mathematics, Nanjing University of Information Science and Technology, Nanjing, 210044 People’s Republic of China; 2 Fakultät für Mathematik, Universität Wien, Oskar-Morgenstern-Platz 1, 1090 Vienna, Austria

**Keywords:** Congruences, Binomial coefficients, Domb numbers, Binary quadratic forms, Primary 11A07 Secondary 11B65, 11B83, 11E16

## Abstract

In this paper, we prove two recently conjectured supercongruences (modulo $p^3$, where *p* is any prime greater than 3) of Zhi-Hong Sun on truncated sums involving the Domb numbers. Our proofs involve a number of ingredients such as congruences involving specialized Bernoulli polynomials, harmonic numbers, binomial coefficients, and hypergeometric summations and transformations.

## Introduction

The *Domb numbers* $\{D_n\}$, defined by

$$\begin{aligned} D_n=\sum _{k=0}^n\left( {\begin{array}{c}n\\ k\end{array}}\right) ^2\left( {\begin{array}{c}2k\\ k\end{array}}\right) \left( {\begin{array}{c}2n-2k\\ n-k\end{array}}\right) \end{aligned}$$

for non-negative integers *n*, first appeared in an extensive study by  Domb [4] on interacting particles on crystal lattices. In particular, Domb showed that $D_n$ counts the number of 2*n*-step polygons on the diamond lattice.

The Domb numbers also appear in a variety of other settings, such as in the coefficients in several known series for $1/\pi$. For example, from [2, Equation (1.3)] we know that

$$\begin{aligned} \sum _{n=0}^\infty \frac{5n+1}{64^n}D_n=\frac{8}{\sqrt{3}\pi }. \end{aligned}$$

In [10, Theorem 3.1],  Rogers showed the following generating function for the Domb numbers by applying a rather intricate method:

$$\begin{aligned} \sum _{n=0}^\infty D_nu^n= \frac{1}{1-4u}\sum _{k=0}^\infty \left( {\begin{array}{c}2k\\ k\end{array}}\right) ^2 \left( {\begin{array}{c}3k\\ k\end{array}}\right) \left(\frac{u^2}{(1-4u)^3}\right)^k, \end{aligned}$$

where |*u*| is sufficiently small. Mu and Sun [9, Equation (1.11)] proved a congruence involving the Domb numbers by applying the telescoping method: For any prime $p>3$, we have the supercongruence

$$\begin{aligned} \sum _{k=0}^{p-1}\frac{3k^2+k}{16^k}D_k\equiv -4p^4q_p(2)\quad \ (\mathrm{{mod}}\ p^5), \end{aligned}$$

where $q_p(a)$ denotes the Fermat quotient $(a^{p-1}-1)/p$.

In [5], Liu proved a couple of conjectures of Sun and Sun. In particular he confirmed [5, Theorem 1.3] that for any positive integer *n* the two sums

$$\begin{aligned} \frac{1}{n}\sum _{k=0}^{n-1}(2k+1)D_k8^{n-1-k}\ \ \ \text{ and }\ \ \ \frac{1}{n}\sum _{k=0}^{n-1}(2k+1)D_k(-8)^{n-1-k} \end{aligned}$$

are also positive integers.

Sun [21, Conjecture 4.1] conjectured the following congruence for the Domb numbers: Let $p>3$ be a prime. Then

$$\begin{aligned} D_{p-1}\equiv 64^{p-1}-\frac{p^3}{6}B_{p-3}\quad \ (\mathrm{{mod}}\ p^4), \end{aligned}$$

where $\{B_n\}$ are the Bernoulli numbers given by

$$\begin{aligned} B_0=1,\ \ \ \sum _{k=0}^{n-1}\left( {\begin{array}{c}n\\ k\end{array}}\right) B_{k}=0\ \ (n\ge 2). \end{aligned}$$

This conjecture was confirmed by the first author and  Wang [8]. For more research on Domb numbers, we kindly refer the readers to [5, 7, 14, 19, 22] (and the references therein).

The main result of this paper is Theorem 1.1 which contains two supercongruences that were originally conjectured by Sun in [13, Conjecture 3.5, Conjecture 3.6]. What makes them interesting is that their formulations involve the binary quadratic form $x^2+3y^2$ for primes *p* that are congruent to 1 modulo 3. (It is well-known that any prime $p\equiv 1\ (\mathrm{{mod}}\ 3)$ can be expressed as $p=x^2+3y^2$ for some integers *x* and *y*, an assertion first made by Fermat and subsequently proved by Euler, see [3]. In his paper [13], Sun stated further conjectures of similar type, involving different moduli, and other binary quadratic forms.) First, Sun defined

$$\begin{aligned} R_3(p)=\left( 1+2p+\frac{4}{3}(2^{p-1}-1) -\frac{3}{2}(3^{p-1}-1)\right) \left( {\begin{array}{c}\frac{p-1}{2}\\ \lfloor p/6\rfloor \end{array}}\right) ^2. \end{aligned}$$

The two supercongruences which we will confirm are as follows.

### Theorem 1.1

Let $p>3$ be a prime. Then

$$\begin{aligned}&\sum _{k=0}^{p-1}k^3\frac{D_k}{4^k}\\&\quad \equiv {\left\{ \begin{array}{ll}-\frac{64}{45}x^2+\frac{32}{45}p+\frac{43p^2}{90x^2}\;\ (\mathrm{{mod}}\ p^3) &{} \quad \text {if} \,\,\ \textit{p}=\textit{x}^2+3\textit{y}^2\equiv 1\ (\mathrm{{mod}}\ 3), \\ \frac{28}{9}R_3(p)\;\ (\mathrm{{mod}}\ p^2) &{} \quad \text {if} \,\,\ \textit{p}\equiv 2\ (\mathrm{{mod}}\ 3)\ \text{ and }\ \textit{p}\ne 5,\end{array}\right. }\\&\sum _{k=0}^{p-1}k^3\frac{D_k}{16^k}\\&\equiv {\left\{ \begin{array}{ll}\frac{4}{45}x^2-\frac{2}{45}p+\frac{p^2}{45x^2}\;\ (\mathrm{{mod}}\ p^3) &{}\text {if} \,\,\ \textit{p}=\textit{x}^2+3\textit{y}^2\equiv 1\ (\mathrm{{mod}}\ 3), \\ -\frac{4}{9}R_3(p)\;\ (\mathrm{{mod}}\ p^2) &{}\text {if} \,\, \ \textit{p}\equiv 2\ (\mathrm{{mod}}\ 3).\end{array}\right. } \end{aligned}$$

Our preparations for the proof of this theorem consist of seven lemmas that we give in Sect. 2. These are used in Sect. 3, devoted to the actual proof of Theorem 1.1. As tools for establishing the results in Sects. 2 and 3 we utilize some congruences from [6, 7] and several combinatorial identities that can be found and proved by the package Sigma [11] via the software Mathematica.

## Preliminary lemmas

Recall that the Bernoulli polynomials $\{B_n(x)\}$ are given by

$$\begin{aligned} B_n(x)=\sum _{k=0}^n\left( {\begin{array}{c}n\\ k\end{array}}\right) B_kx^{n-k}\ \ (n=0,1,2,\ldots ), \end{aligned}$$

where, as before, $\{B_n\}$ are the Bernoulli numbers. We will also use the classical Legendre symbol $\big (\frac{a}{q}\big )$ (for integer *a* and odd prime *q*). The following lemma involving the (generalized) harmonic numbers can be easily deduced from [16, Theorem 5.2 (c)], [17, Theorem 3.9 (ii), (iii), (iv)], [17, third equation on p. 302], and the simple identity

$$\begin{aligned} \sum _{1\le k<\frac{2p}{3}}\frac{1}{k}= \sum _{1\le k<\frac{2p}{3}}\frac{(-1)^{k-1}}{k} +\sum _{1\le k<\frac{p}{3}}\frac{1}{k}. \end{aligned}$$

### Lemma 2.1

Let $p>5$ be a prime. Then

$$\begin{aligned} H_{\frac{p-1}{2}}&\equiv -2q_p(2)\;\ (\mathrm{{mod}}\ p),\\ H_{\lfloor \frac{p}{6}\rfloor }&\equiv -2q_p(2)-\frac{3}{2}q_p(3)\;\ (\mathrm{{mod}}\ p),\\ H_{\lfloor \frac{p}{3}\rfloor }^{(2)}&\equiv \frac{1}{2}\left( \frac{p}{3}\right) B_{p-2}\Big (\frac{1}{3}\Big )\;\ (\mathrm{{mod}}\ p),\\ H_{\lfloor \frac{p}{3}\rfloor }&\equiv -\frac{3}{2}q_p(3)+\frac{3p}{4}q^2_p(3)-\frac{p}{6}\left( \frac{p}{3}\right) B_{p-2} \Big (\frac{1}{3}\Big )\;\ (\mathrm{{mod}}\ p^2),\\ H_{\lfloor \frac{2p}{3}\rfloor }&\equiv -\frac{3}{2}q_p(3)+\frac{3p}{4}q^2_p(3)+\frac{p}{3}\left( \frac{p}{3}\right) B_{p-2} \Big (\frac{1}{3}\Big )\;\ (\mathrm{{mod}}\ p^2). \end{aligned}$$

The above lemma can be compared to results established in [18], which contains similar congruences involving the Bernoulli polynomials but, rather than for the harmonic numbers $\{H_n\}$, for the special numbers $\{U_n\}$, which in [18] were recursively defined by

$$\begin{aligned} U_0=1,\ \ \ U_n=-2\sum _{k=1}^{\lfloor n/2\rfloor }\left( {\begin{array}{c}n\\ 2k\end{array}}\right) U_{n-2k}\ \ \ (n\ge 1). \end{aligned}$$

### Lemma 2.2

([7, Lemma 2.2]) Let $p>5$ be a prime. If $0\le j\le (p-1)/2$, then we have

$$\begin{aligned} \left( {\begin{array}{c}3j\\ j\end{array}}\right) \left( {\begin{array}{c}p+j\\ 3j+1\end{array}}\right) \equiv \frac{p}{3j+1}(1-pH_{2j}+pH_j)\;\ (\mathrm{{mod}}\ p^3). \end{aligned}$$

### Lemma 2.3

([7, Lemma 2.3]) Let $p>3$ be a prime. For any *p*-adic integer *t*, we have

$$\begin{aligned} \left( {\begin{array}{c}\frac{2p-2}{3}+pt\\ \frac{p-1}{2}\end{array}}\right) \equiv \left( {\begin{array}{c}\frac{2p-2}{3}\\ \frac{p-1}{2}\end{array}}\right) \left( 1+pt(H_{\frac{2p-2}{3}}-H_{\frac{p-1}{6}})\right) \;\ (\mathrm{{mod}}\ p^2). \end{aligned}$$

### Lemma 2.4

Let $p>3$ be a prime. If $p=x^2+3y^2\equiv 1\ (\mathrm{{mod}}\ 3)$, then

$$\begin{aligned} p\sum _{k=0}^{\frac{p-1}{2}}\frac{\left( {\begin{array}{c}2k\\ k\end{array}}\right) ^2}{(3k+4)16^k} \equiv \frac{4}{25}\left( 4x^2-2p-\frac{p^2}{4x^2}\right) \;\ (\mathrm{{mod}}\ p^3). \end{aligned}$$

### Proof

By using Sigma, we establish the following identity:

$$\begin{aligned} \sum _{k=0}^n\frac{\left( {\begin{array}{c}n\\ k\end{array}}\right) \left( {\begin{array}{c}n+k\\ k\end{array}}\right) (-1)^k}{3k+4}=-\frac{1}{(3n-1)(3n+1)(3n+4)}\prod _{k=1}^n\frac{3k-1}{3k-2}. \end{aligned}$$

(In terms of classical identities for hypergeometric series, this evaluation is equivalent to the $(a,b,c)\mapsto (n+1,4/3,1)$ case of the Pfaff–Saalschütz summation [12, Appendix III, Equation (III.2)].) So modulo $p^3$, we have

$$\begin{aligned}&p\sum _{k=0}^{\frac{p-1}{2}}\frac{\left( {\begin{array}{c}2k\\ k\end{array}}\right) ^2}{(3k+4)16^k}\\&\equiv p\sum _{k=0}^{\frac{p-1}{2}}\frac{\left( {\begin{array}{c}\frac{p-1}{2}\\ k\end{array}}\right) \left( {\begin{array}{c}\frac{p-1}{2}+k\\ k\end{array}}\right) (-1)^k}{3k+4}+\left( {\begin{array}{c}-\frac{1}{2}\\ \frac{p-4}{3}\end{array}}\right) ^2+\left( {\begin{array}{c}\frac{p-1}{2}\\ \frac{p-4}{3}\end{array}}\right) \left( {\begin{array}{c}\frac{p-1}{2}+\frac{p-4}{3}\\ \frac{p-4}{3}\end{array}}\right) \\&=\frac{4}{25-9p^2}\frac{(\frac{2}{3})_{\frac{p-1}{2}}}{(\frac{1}{3})_{\frac{p-1}{3}}(\frac{p}{3}+1)_{\frac{p-1}{6}}}+\left( {\begin{array}{c}-\frac{1}{2}\\ \frac{p-4}{3}\end{array}}\right) ^2+\left( {\begin{array}{c}\frac{p-1}{2}\\ \frac{p-4}{3}\end{array}}\right) \left( {\begin{array}{c}\frac{p-1}{2}+\frac{p-4}{3}\\ \frac{p-4}{3}\end{array}}\right) , \end{aligned}$$

where we used the standard notation for the shifted factorial $(a)_n=\prod _{j=0}^{n-1}(a+j)$ (cf. [12, Section 1.1.1]). It is easy to check that

2.1 $$\begin{aligned} \left( {\begin{array}{c}-\frac{1}{2}\\ \frac{p-4}{3}\end{array}}\right) ^2&=\frac{4(p-1)^2}{(2p-5)^2}\left( {\begin{array}{c}-\frac{1}{2}\\ \frac{p-1}{3}\end{array}}\right) ^2,\nonumber \\ \left( {\begin{array}{c}\frac{p-1}{2}\\ \frac{p-4}{3}\end{array}}\right) \left( {\begin{array}{c}\frac{p-1}{2}+\frac{p-4}{3}\\ \frac{p-4}{3}\end{array}}\right)&=\frac{4(p-1)}{5(p+5)}\left( {\begin{array}{c}\frac{p-1}{2}\\ \frac{p-1}{3}\end{array}}\right) \left( {\begin{array}{c}\frac{p-1}{2}+\frac{p-1}{3}\\ \frac{p-1}{3}\end{array}}\right) . \end{aligned}$$

These identities, together with [6, pp. 14], yield

$$\begin{aligned}&p\sum _{k=0}^{\frac{p-1}{2}}\frac{\left( {\begin{array}{c}2k\\ k\end{array}}\right) ^2}{(3k+4)16^k}\\&\equiv \frac{4}{25-9p^2}\frac{(\frac{2}{3})_{\frac{p-1}{2}}}{(\frac{1}{3})_{\frac{p-1}{3}}(\frac{p}{3}+1)_{\frac{p-1}{6}}}+\frac{4(p-1)^2}{(2p-5)^2} \left( {\begin{array}{c}-\frac{1}{2}\\ \frac{p-1}{3}\end{array}}\right) ^2\\&\quad \;+\frac{4(p-1)}{5(p+5)}\left( {\begin{array}{c}\frac{p-1}{2}\\ \frac{p-1}{3}\end{array}}\right) \left( {\begin{array}{c}\frac{p-1}{2}+\frac{p-1}{3}\\ \frac{p-1}{3}\end{array}}\right) \\&\equiv \frac{4}{25}\left( 1+\frac{9p^2}{25}\right) (-1)^{\frac{p-1}{6}}\left( {\begin{array}{c}\frac{p-1}{2}\\ \frac{p-1}{3}\end{array}}\right) \left( {\begin{array}{c}\frac{2p-2}{3}\\ \frac{p-1}{2}\end{array}}\right) \\&\quad \;\times \left( 1-\frac{2p}{3}q_p(2)+\frac{5p^2}{9}q^2_p(2) +\frac{5p^2}{12}\left( \frac{p}{3}\right) B_{p-2}\Big (\frac{1}{3}\Big )\right) \\&\quad \;+\frac{4}{25}\left( 1-\frac{6p}{5}-\frac{3p^2}{25}\right) \left( {\begin{array}{c}\frac{p-1}{2}\\ \frac{p-1}{3}\end{array}}\right) ^2\\&\quad \;\;\times \left( 1-\frac{3p}{2}q_p(3)+\frac{15p^2}{8}q^2_p(3)+\frac{5p^2}{24}\left( \frac{p}{3}\right) B_{p-2}\Big (\frac{1}{3}\Big )\right) \\&\quad \;-\frac{4}{25}\left( 1-\frac{6p}{5}+\frac{6p^2}{25}\right) \left( {\begin{array}{c}\frac{p-1}{2}\\ \frac{p-1}{3}\end{array}}\right) \left( {\begin{array}{c}\frac{5p-5}{6}\\ \frac{p-1}{3}\end{array}}\right) \quad \ (\mathrm{{mod}}\ p^3). \end{aligned}$$

Again, by [6, pp. 14–15], we have

$$\begin{aligned}&p\sum _{k=0}^{\frac{p-1}{2}}\frac{\left( {\begin{array}{c}2k\\ k\end{array}}\right) ^2}{(3k+4)16^k} \\ {}&\equiv \frac{4}{25}\left( 1+\frac{9p^2}{25}\right) \left( 4x^2-2p-\frac{p^2}{4x^2}\right) \\&\quad \;\times \left( 1+\frac{2p}{3}q_p(2)-\frac{p^2}{9}q^2_p(2) +\frac{5p^2}{24}\left( \frac{p}{3}\right) B_{p-2}\Big (\frac{1}{3}\Big )\right) \\&\quad \;\times \left( 1-\frac{2p}{3}q_p(2)+\frac{5p^2}{9}q^2_p(2) +\frac{5p^2}{12}\left( \frac{p}{3}\right) B_{p-2}\Big (\frac{1}{3}\Big )\right) \\&\quad \;+\frac{4}{25}\left( 1-\frac{6p}{5}-\frac{3p^2}{25}\right) \left( 4x^2-2p-\frac{p^2}{4x^2}\right) \\&\quad \;\;\times \bigg (1-\frac{3p}{2}q_p(3) +\frac{15p^2}{8}q^2_p(3)+\frac{5p^2}{24}\left( \frac{p}{3}\right) B_{p-2}\Big (\frac{1}{3}\Big )\bigg )\\&\quad \;\;\times \bigg (1-\frac{4p}{3}q_p(2)+\frac{3p}{2}q_p(3) +\frac{14p^2}{9}q^2_p(2)-2p^2q_p(2)q_p(3)\\&\quad +\frac{3p^2}{8}q^2_p(3) +\frac{p^2}{8}\left( \frac{p}{3}\right) B_{p-2} \Big (\frac{1}{3}\Big )\bigg )\\&\quad \;-\frac{4}{25}\left( 1-\frac{6p}{5}+\frac{6p^2}{25}\right) \left( 4x^2-2p-\frac{p^2}{4x^2}\right) \\&\quad \;\;\times \left( 1-\frac{4p}{3}q_p(2)+\frac{14p^2}{9}q^2_p(2) +\frac{23p^2}{24}\left( \frac{p}{3}\right) B_{p-2} \Big (\frac{1}{3}\Big )\right) \;\ (\mathrm{{mod}}\ p^3). \end{aligned}$$

It is easy to check that the right-side of the above congruence is congruent to $\frac{4}{25}\left( 4x^2-2p-\frac{p^2}{4x^2}\right)$ modulo $p^3$. Therefore we immediately get the desired result stated in Lemma 2.4. $\square$

### Lemma 2.5

Let $p>3$ be a prime with $p=x^2+3y^2\equiv 1\ (\mathrm{{mod}}\ 3)$ and let $k=(p-4)/3$. Then

$$\begin{aligned}&\big (k(k+1)(k+3)+(2-k^2)(3k+1)p-(k+2)(3k+1)(3k+2)p^2\big )\\&\qquad \times \frac{\left( {\begin{array}{c}-\frac{1}{2}\\ k\end{array}}\right) ^2}{(k+1)(3k+2)} \left( \frac{\left( {\begin{array}{c}3k\\ k\end{array}}\right) \left( {\begin{array}{c}p+k\\ 3k+1\end{array}}\right) }{3k+4} -\frac{1-pH_{2k}+pH_k}{3k+1}\right) \\&\qquad \quad \equiv -\frac{184p^2x^2}{125}\;\ (\mathrm{{mod}}\ p^3) \end{aligned}$$

and

$$\begin{aligned}&\big (k(1+2k)+2p(k+1)(3k+1)-2p^2(3k+1)(3k+2)\big )\\&\qquad \times \frac{\left( {\begin{array}{c}-\frac{1}{2}\\ k\end{array}}\right) ^2}{3k+2} \left( \frac{\left( {\begin{array}{c}3k\\ k\end{array}}\right) \left( {\begin{array}{c}p+2k\\ 3k+1\end{array}}\right) }{3k+4} +\frac{1+pH_{2k}-pH_k}{3k+1}\right) \\&\quad \qquad \equiv -\frac{184p^2x^2}{125}\;\ (\mathrm{{mod}}\ p^3). \end{aligned}$$

### Proof

It is easy to see that

$$\begin{aligned}&\frac{\left( {\begin{array}{c}3k\\ k\end{array}}\right) \left( {\begin{array}{c}p+k\\ 3k+1\end{array}}\right) }{3k+4}-\frac{1-pH_{2k}+pH_k}{3k+1}\\&\quad =\left( {\begin{array}{c}3k+3\\ k+1\end{array}}\right) \left( {\begin{array}{c}p+k+1\\ 3k+4\end{array}}\right) \frac{2(2p-5)(p-1)^2}{(4p-1)(p-3)(p+2)(p+5)}\\&\qquad \;-\frac{1-pH_{2k+2}+pH_{k+1}+\frac{p}{2k+2}+\frac{p}{2k+1}-\frac{p}{k+1}}{3k+1}. \end{aligned}$$

By Lemma 2.2 we have

$$\begin{aligned} \left( {\begin{array}{c}3k+3\\ k+1\end{array}}\right) \left( {\begin{array}{c}p+k+1\\ 3k+4\end{array}}\right) \equiv 1-pH_{2k+2}+pH_{k+1}\ (\mathrm{{mod}}\ p^3). \end{aligned}$$

Thus,

$$\begin{aligned} \frac{\left( {\begin{array}{c}3k\\ k\end{array}}\right) \left( {\begin{array}{c}p+k\\ 3k+1\end{array}}\right) }{3k+4}-\frac{1-pH_{2k} +pH_k}{3k+1}\equiv -\frac{207p^2}{100}\;\ (\mathrm{{mod}}\ p^3). \end{aligned}$$

Together with (2.1) and $$\left( {\begin{array}{c}(p-1)/2\\ (p-1)/3\end{array}}\right) \equiv 2x\ (\mathrm{{mod}}\ p)$$ (cf. [23]), this yields

$$\begin{aligned}&\big (k(k+1)(k+3)+(2-k^2)(3k+1)p-(k+2)(3k+1)(3k+2)p^2\big )\\&\quad \times \frac{\left( {\begin{array}{c}-\frac{1}{2}\\ k\end{array}}\right) ^2}{(k+1)(3k+2)} \left( \frac{\left( {\begin{array}{c}3k\\ k\end{array}}\right) \left( {\begin{array}{c}p+k\\ 3k+1\end{array}}\right) }{3k+4} -\frac{1-pH_{2k}+pH_k}{3k+1}\right) \\&\qquad \equiv -\frac{184p^2x^2}{125}\quad \ (\mathrm{{mod}}\ p^3), \end{aligned}$$

which completes the proof of the first congruence in Lemma 2.5. The proof of the second congruence is similar and therefore omitted. $\square$

### Lemma 2.6

Let $p>2$ be a prime. If $0\le j\le (p-1)/2$, then we have

$$\begin{aligned} \left( {\begin{array}{c}3j\\ j\end{array}}\right) \left( {\begin{array}{c}p+2j\\ 3j+1\end{array}}\right) \equiv \frac{p(-1)^j}{3j+1}(1+pH_{2j}-pH_j)\;\ (\mathrm{{mod}}\ p^3). \end{aligned}$$

If $$(p+1)/2\le j\le p-1$$, then

$$\begin{aligned} \left( {\begin{array}{c}3j\\ j\end{array}}\right) \left( {\begin{array}{c}p+2j\\ 3j+1\end{array}}\right) \equiv \frac{2p(-1)^j}{3j+1}\;\ (\mathrm{{mod}}\ p^2). \end{aligned}$$

### Proof

If $0\le j\le (p-1)/2$, we can get the result from [15, pp. 24–25]. If $(p+1)/2\le j\le p-1$, then

$$\begin{aligned} \left( {\begin{array}{c}3j\\ j\end{array}}\right) \left( {\begin{array}{c}p+2j\\ 3j+1\end{array}}\right)&=\frac{(p+2j)\cdots (2p+1)(2p)(2p-1)\cdots (p+1)p(p-1)\cdots (p-j)}{(3j+1)j!(2j)!}\\&\equiv \frac{2p^2(2j)\cdots (p+1)(p-1)!(-1)^{j}(j)!}{(3j+1)j!(2j)!}\equiv \frac{2p(-1)^j}{3j+1}\;\ (\mathrm{{mod}}\ p^2), \end{aligned}$$

which completes the proof of Lemma 2.6. $\square$

### Lemma 2.7

Let $p>3$ be a prime with $p=x^2+3y^2\equiv 1\ (\mathrm{{mod}}\ 3)$. Then

$$\begin{aligned} p\sum _{j=0}^{\frac{p-1}{2}}\frac{\left( {\begin{array}{c}2j\\ j\end{array}}\right) ^2(H_{2j}-H_j)}{(3j+4)16^j}\equiv -\frac{18}{125}(4x^2-2p)\;\ (\mathrm{{mod}}\ p^2). \end{aligned}$$

### Proof

By using Sigma, we establish the following identity:

$$\begin{aligned}&\sum _{j=0}^n\frac{\left( {\begin{array}{c}n\\ j\end{array}}\right) \left( {\begin{array}{c}n+j\\ j\end{array}}\right) (-1)^j(H_{2j}-H_j)}{3j+4} =-\frac{9(2n+1)}{10(3n-1)(3n+4)}\\&\quad +\frac{(\frac{2}{3})_n}{(3n-1)(3n+1)(3n+4)(\frac{1}{3})_n}\left( \frac{9}{10} +\sum _{k=1}^n\frac{(\frac{1}{3})_k}{k(\frac{2}{3})_k}\right) . \end{aligned}$$

Substituting $n=(p-1)/2$ into the above identity, then modulo $p^2$ we have

$$\begin{aligned} p\sum _{j=0}^{\frac{p-1}{2}}\frac{\left( {\begin{array}{c}2j\\ j\end{array}}\right) ^2(H_{2j}-H_j)}{(3j+4)16^j}\equiv \frac{p(\frac{2}{3})_{\frac{p-1}{2}}}{\frac{3p-5}{2}\frac{3p-1}{2}\frac{3p+5}{2}(\frac{1}{3})_{\frac{p-1}{2}}}\left( \frac{9}{10}+\sum _{k=1}^{\frac{p-1}{2}}\frac{(\frac{1}{3})_k}{k(\frac{2}{3})_k}\right) . \end{aligned}$$

In view of [7, pp. 9] and [6, pp. 14–15], we have

$$\begin{aligned} \frac{(\frac{2}{3})_{\frac{p-1}{2}}}{(\frac{1}{3})_{\frac{p-1}{3}}(\frac{p}{3}+1)_{\frac{p-1}{6}}}&\equiv 4x^2-2p\quad \ (\mathrm{{mod}}\ p^2),\\ \frac{(\frac{2}{3})_{\frac{p-1}{2}}}{(\frac{1}{3})_{\frac{p-1}{2}}} \sum _{k=1}^{\frac{p-1}{2}}\frac{(\frac{1}{3})_k}{k(\frac{2}{3})_k}&\equiv 0\quad \ (\mathrm{{mod}}\ p). \end{aligned}$$

Hence,

$$\begin{aligned} p\sum _{j=0}^{\frac{p-1}{2}}\frac{\left( {\begin{array}{c}2j\\ j\end{array}}\right) ^2(H_{2j}-H_j)}{(3j+4)16^j}&\equiv \frac{9}{10}\frac{p(\frac{2}{3})_{\frac{p-1}{2}}}{\frac{3p-5}{2}\frac{3p-1}{2}\frac{3p+5}{2}(\frac{1}{3})_{\frac{p-1}{2}}}\\&\equiv -\frac{9}{10}\frac{4}{25}(4x^2-2p)=-\frac{18}{125}(4x^2-2p)\quad \ (\mathrm{{mod}}\ p^2). \end{aligned}$$

The proof of Lemma 2.7 is complete. $\square$

## Proof of Theorem 1.1

Our proof of Theorem 1.1 heavily relies on the following two transformation formulas due to  Chan and Zudilin [1] and  Sun [19] respectively,

3.1 $$\begin{aligned} \sum _{k=0}^n\left( {\begin{array}{c}n\\ k\end{array}}\right) ^2\left( {\begin{array}{c}2k\\ k\end{array}}\right) \left( {\begin{array}{c}2n-2k\\ n-k\end{array}}\right) =\sum _{k=0}^{n}(-1)^k\left( {\begin{array}{c}n+2k\\ 3k\end{array}}\right) \left( {\begin{array}{c}2k\\ k\end{array}}\right) ^2\left( {\begin{array}{c}3k\\ k\end{array}}\right) 16^{n-k}, \end{aligned}$$

3.2 $$\begin{aligned} \sum _{k=0}^n\left( {\begin{array}{c}n\\ k\end{array}}\right) ^2\left( {\begin{array}{c}2k\\ k\end{array}}\right) \left( {\begin{array}{c}2n-2k\\ n-k\end{array}}\right) =\sum _{k=0}^{\lfloor n/2\rfloor }\left( {\begin{array}{c}n+k\\ 3k\end{array}}\right) \left( {\begin{array}{c}2k\\ k\end{array}}\right) ^2\left( {\begin{array}{c}3k\\ k\end{array}}\right) 4^{n-2k}. \end{aligned}$$

### Proof of Theorem 1.1

We first consider the first congruence in Theorem 1.1 in the case $p=x^2+3y^2\equiv 1\ (\mathrm{{mod}}\ 3)$. By (3.2), we have

$$\begin{aligned} \sum _{k=0}^{p-1}k^3\frac{D_k}{4^k}&=\sum _{k=0}^{p-1}\frac{k^3}{4^k}\sum _{j=0}^{\lfloor k/2\rfloor } \left( {\begin{array}{c}k+j\\ 3j\end{array}}\right) \left( {\begin{array}{c}2j\\ j\end{array}}\right) ^2\left( {\begin{array}{c}3j\\ j\end{array}}\right) 4^{k-2j}\\&=\sum _{j=0}^{(p-1)/2}\frac{\left( {\begin{array}{c}2j\\ j\end{array}}\right) ^2\left( {\begin{array}{c}3j\\ j\end{array}}\right) }{16^j}\sum _{k=2j}^{p-1}k^3\left( {\begin{array}{c}k+j\\ 3j\end{array}}\right) . \end{aligned}$$

By using Sigma, we establish the following identity:

$$\begin{aligned} \sum _{k=2j}^{n-1}k^3\left( {\begin{array}{c}k+j\\ 3j\end{array}}\right) =\frac{\sigma _1}{(j+1)(3j+2)(3j+4)}\left( {\begin{array}{c}n+j\\ 3j+1\end{array}}\right) , \end{aligned}$$

where

$$\begin{aligned} \sigma _1&=j(j+1)(j+3)+n(2-j^2)(3j+1)-n^2(j+2)(3j+1)(3j+2)\\&\quad \;+n^3(j+1)(3j+1)(3j+2). \end{aligned}$$

Thus,

$$\begin{aligned} \sum _{k=0}^{p-1}k^3\frac{D_k}{4^k} =\sum _{j=0}^{\frac{p-1}{2}}\frac{\left( {\begin{array}{c}2k\\ k\end{array}}\right) ^2\left( {\begin{array}{c}3j\\ j\end{array}}\right) \left( {\begin{array}{c}p+j\\ 3j+1\end{array}}\right) }{16^k}\frac{\sigma _1}{(j+1)(3j+2)(3j+4)}. \end{aligned}$$

Let

$$\begin{aligned} \sigma _2=k(k+1)(k+3)+p(2-k^2)(3k+1)-p^2(k+2)(3k+1)(3k+2). \end{aligned}$$

In view of Lemma 2.2, we have for $p\equiv 1\ (\mathrm{{mod}}\ 3)$ the supercongruence

$$\begin{aligned} \sum _{k=0}^{p-1}k^3\frac{D_k}{4^k}\nonumber&\equiv \sum _{k=0}^{\frac{p-1}{2}} \frac{\left( {\begin{array}{c}2k\\ k\end{array}}\right) ^2\left( {\begin{array}{c}3k\\ k\end{array}}\right) \left( {\begin{array}{c}p+k\\ 3k+1\end{array}}\right) }{16^k}\frac{\sigma _2}{(k+1)(3k+2)(3k+4)}\\&\equiv \sum _{k=0}^{\frac{p-1}{2}}\frac{\left( {\begin{array}{c}2k\\ k\end{array}}\right) ^2}{16^k}\frac{p(1-pH_{2k} +pH_k)\sigma _2}{(k+1)(3k+1)(3k+2)(3k+4)}+S_1\quad \ (\mathrm{{mod}}\ p^3), \end{aligned}$$

where $S_1$ is defined by the following expression with $k=(p-4)/3$,

$$\begin{aligned} S_1&=\big (k(k+1)(k+3)+(2-k^2)(3k+1)p-(k+2)(3k+1)(3k+2)p^2\big )\\&\quad \;\times \frac{\left( {\begin{array}{c}-\frac{1}{2}\\ k\end{array}}\right) ^2}{(k+1)(3k+2)}\left( \frac{\left( {\begin{array}{c}3k\\ k\end{array}}\right) \left( {\begin{array}{c}p+k\\ 3k+1\end{array}}\right) }{3k+4} -\frac{1-pH_{2k}+pH_k}{3k+1}\right) . \end{aligned}$$

In view of [20, Equation (12)] and [7, Equation (2.10)], we have

3.3 $$\begin{aligned} \sum _{k=0}^{\frac{p-1}{2}}\frac{\left( {\begin{array}{c}2k\\ k\end{array}}\right) ^2}{(k+1)16^k}\equiv 0\ (\mathrm{{mod}}\ p^2),\end{aligned}$$

3.4 $$\begin{aligned} \sum _{k=0}^{\frac{p-1}{2}}\frac{\left( {\begin{array}{c}2k\\ k\end{array}}\right) ^2(H_{2k}-H_k)}{(3k+1)16^k} \equiv 0\ (\mathrm{{mod}}\ p). \end{aligned}$$

And in view of [7, pp. 13–14], we have

3.5 $$\begin{aligned} \frac{2p^2}{3}\sum _{k=0}^{\frac{p-1}{2}}\frac{\left( {\begin{array}{c}2k\\ k\end{array}}\right) ^2(H_{2k}-H_k)}{(3k+2)16^k} \equiv \sum _{k=0}^{\frac{p-1}{2}}\frac{p\left( {\begin{array}{c}2k\\ k\end{array}}\right) ^2}{(3k+2)16^k}\equiv -\frac{p^2}{x^2}\ (\mathrm{{mod}}\ p^3). \end{aligned}$$

Hence by (3.5), Lemma 2.5 and [6, Theorem 1.2], we have

$$\begin{aligned} \sum _{k=0}^{p-1}k^3\frac{D_k}{4^k}+\frac{184p^2x^2}{125}&\equiv \frac{p}{27} \sum _{k=0}^{\frac{p-1}{2}}\frac{\left( {\begin{array}{c}2k\\ k\end{array}}\right) ^2}{16^k}\left( \frac{-8}{3k+1} +\frac{21}{3k+2}-\frac{10}{3k+4}\right) \\&\quad \;+\frac{p^2}{3}\sum _{k=0}^{\frac{p-1}{2}}\frac{\left( {\begin{array}{c}2k\\ k\end{array}}\right) ^2}{16^k} \left( \frac{-3}{k+1}+\frac{7}{3k+2}+\frac{1}{3k+4}\right) \\&\quad \;-p^3\sum _{k=0}^{\frac{p-1}{2}}\frac{\left( {\begin{array}{c}2k\\ k\end{array}}\right) ^2}{16^k} \left( \frac{1}{k+1}-\frac{2}{3k+4}\right) \\&\quad \;-\frac{p^2}{27}\sum _{k=0}^{\frac{p-1}{2}}\frac{\left( {\begin{array}{c}2k\\ k\end{array}}\right) ^2(H_{2k}-H_k)}{16^k}\left( \frac{-8}{3k+1}+\frac{21}{3k+2}-\frac{10}{3k+4}\right) \\&\quad \;-\frac{p^3}{3}\sum _{k=0}^{\frac{p-1}{2}}\frac{\left( {\begin{array}{c}2k\\ k\end{array}}\right) ^2 (H_{2k}-H_k)}{16^k}\left( \frac{-3}{k+1}+\frac{7}{3k+2}+\frac{1}{3k+4}\right) \\&\equiv \left( -\frac{8}{27}{-}\frac{10}{27}\frac{4}{25}\right) \left( 4x^2{-}2p{-}\frac{p^2}{4x^2}\right) {-}\frac{21}{27}\frac{p^2}{x^2}{+}\frac{p}{3}\frac{4}{25}(4x^2-2p)\\&\quad \;+2p^2\frac{16x^2}{25}-\frac{10p}{27}\frac{18}{125} (4x^2-2p)+\frac{21}{27}\frac{3}{2}\frac{p^2}{x^2}+\frac{p^2}{3}\frac{18}{125}4x^2\\&\equiv -\frac{64x^2}{45}+\frac{32p}{45}+\frac{43p^2}{90x^2}+\frac{184p^2x^2}{125}\quad \ (\mathrm{{mod}}\ p^3). \end{aligned}$$

Thus we immediately obtain the desired result

3.6 $$\begin{aligned} \sum _{k=0}^{p-1}k^3\frac{D_k}{4^k}\equiv -\frac{64x^2}{45}+ \frac{32p}{45}+\frac{43p^2}{90x^2}\quad \ (\mathrm{{mod}}\ p^3). \end{aligned}$$

Now we are ready to prove the case $p\equiv 2\ (\mathrm{{mod}}\ 3)$ with $p>5$. As before,

$$\begin{aligned} \sum _{k=0}^{p-1}k^3\frac{D_k}{4^k}\nonumber&\equiv \sum _{k=0}^{\frac{p-1}{2}} \frac{\left( {\begin{array}{c}2k\\ k\end{array}}\right) ^2\left( {\begin{array}{c}3k\\ k\end{array}}\right) \left( {\begin{array}{c}p+k\\ 3k+1\end{array}}\right) }{16^k}\frac{k(k+1)(k+3) +p(2-k^2)(3k+1)}{(k+1)(3k+2)(3k+4)}\\&\equiv \sum _{k=0}^{\frac{p-1}{2}}\frac{\left( {\begin{array}{c}2k\\ k\end{array}}\right) ^2}{16^k} \frac{p(1-pH_{2k}+pH_k)(k(k+1)(k+3)+p(2-k^2)(3k+1))}{(k+1)(3k+1)(3k+2)(3k+4)}\\&\equiv \frac{p}{27}\sum _{k=0}^{\frac{p-1}{2}}\frac{\left( {\begin{array}{c}2k\\ k\end{array}}\right) ^2}{16^k} \left( \frac{-8}{3k+1}+\frac{21}{3k+2}-\frac{10}{3k+4}\right) \\&\quad \;+\frac{p^2}{3}\sum _{k=0}^{\frac{p-1}{2}}\frac{\left( {\begin{array}{c}2k\\ k\end{array}}\right) ^2}{16^k} \left( \frac{-3}{k+1}+\frac{7}{3k+2}+\frac{1}{3k+4}\right) \\&\quad \;-\frac{p^2}{27}\sum _{k=0}^{\frac{p-1}{2}}\frac{\left( {\begin{array}{c}2k\\ k\end{array}}\right) ^2(H_{2k}-H_k)}{16^k}\left( \frac{-8}{3k+1}+\frac{21}{3k+2}-\frac{10}{3k+4}\right) \;\ (\mathrm{{mod}}\ p^2). \end{aligned}$$

In view of [7, pp. 9–10], we have

$$\begin{aligned} \sum _{k=0}^{\frac{p-1}{2}}\frac{\left( {\begin{array}{c}2k\\ k\end{array}}\right) ^2}{(3k+1)16^k}\equiv 0\quad \ (\mathrm{{mod}}\ p), \end{aligned}$$

and in view of Lemma 2.4, we have

$$\begin{aligned} \sum _{k=0}^{\frac{p-1}{2}}\frac{\left( {\begin{array}{c}2k\\ k\end{array}}\right) ^2}{(3k+4)16^k}\equiv 0\quad \ (\mathrm{{mod}}\ p). \end{aligned}$$

Thus,

$$\begin{aligned} \sum _{k=0}^{p-1}k^3\frac{D_k}{4^k}&\equiv \frac{7p}{9}\sum _{k=0}^{\frac{p-1}{2}} \frac{\left( {\begin{array}{c}2k\\ k\end{array}}\right) ^2}{(3k+2)16^k}+\frac{7p^2}{3} \sum _{k=0}^{\frac{p-1}{2}}\frac{\left( {\begin{array}{c}2k\\ k\end{array}}\right) ^2}{(3k+2)16^k}\\&\quad \;-\frac{7p^2}{9}\sum _{k=0}^{\frac{p-1}{2}} \frac{\left( {\begin{array}{c}2k\\ k\end{array}}\right) ^2(H_{2k}-H_k)}{(3k+2)16^k}\quad \ (\mathrm{{mod}}\ p^2). \end{aligned}$$

In view of [7, Equation (4.2)], we have

3.7 $$\begin{aligned} \sum _{k=0}^{\frac{p-1}{2}}\frac{\left( {\begin{array}{c}2k\\ k\end{array}}\right) ^2(H_{2k}-H_k)}{16^k(3k+2)} \equiv 3\sum _{k=0}^{\frac{p-1}{2}}\frac{\left( {\begin{array}{c}2k\\ k\end{array}}\right) ^2}{16^k(3k+2)}\quad \ (\mathrm{{mod}}\ p), \end{aligned}$$

and

3.8 $$\begin{aligned} p\sum _{k=0}^{\frac{p-1}{2}}\frac{\left( {\begin{array}{c}2k\\ k\end{array}}\right) ^2}{(3k+2)16^k} \equiv 4R_3(p)\quad \ (\mathrm{{mod}}\ p^2). \end{aligned}$$

Hence

3.9 $$\begin{aligned} \sum _{k=0}^{p-1}k^3\frac{D_k}{4^k}\equiv \frac{28}{9}R_3(p)\quad \ (\mathrm{{mod}}\ p^2). \end{aligned}$$

Now we consider the other congruences in Theorem 1.1. Similar to above, by (3.1), we have

$$\begin{aligned} \sum _{k=0}^{p-1}k^3\frac{D_k}{16^k}&=\sum _{k=0}^{p-1}\frac{k^3}{16^k} \sum _{j=0}^{k}(-1)^j\left( {\begin{array}{c}k+2j\\ 3j\end{array}}\right) \left( {\begin{array}{c}2j\\ j\end{array}}\right) ^2\left( {\begin{array}{c}3j\\ j\end{array}}\right) 16^{k-j}\\&=\sum _{j=0}^{p-1}\frac{\left( {\begin{array}{c}2j\\ j\end{array}}\right) ^2\left( {\begin{array}{c}3j\\ j\end{array}}\right) }{(-16)^j}\sum _{k=j}^{p-1}k^3\left( {\begin{array}{c}k+2j\\ 3j\end{array}}\right) . \end{aligned}$$

By using Sigma, we establish the following identity:

$$\begin{aligned} \sum _{k=j}^{n-1}k^3\left( {\begin{array}{c}k+2j\\ 3j\end{array}}\right) =\frac{\sigma _3}{(3j+2)(3j+4)}\left( {\begin{array}{c}n+2j\\ 3j+1\end{array}}\right) , \end{aligned}$$

where

$$\begin{aligned} \sigma _3=j(1+2j)+2n(j+1)(3j+1)-2n^2(3j+1)(3j+2)+n^3(3j+1)(3j+2). \end{aligned}$$

Let

$$\begin{aligned} \sigma _4=j(1+2j)+2p(j+1)(3j+1)-2p^2(3j+1)(3j+2). \end{aligned}$$

Thus, if $p\equiv 1\ (\mathrm{{mod}}\ 3)$, then by Lemma 2.6, we have, modulo $p^3$,

$$\begin{aligned}&\sum _{k=0}^{p-1}k^3\frac{D_k}{16^k}-\frac{1}{18p(p+1)}\left( {\begin{array}{c}-1/2\\ \frac{2p-2}{3}\end{array}}\right) ^2 \left( {\begin{array}{c}2p-2\\ \frac{2p-2}{3}\end{array}}\right) \left( {\begin{array}{c}p+\frac{4p-4}{3}\\ 2p-1\end{array}}\right) \\&\quad \equiv \sum _{j=0}^{\frac{p-1}{2}}\frac{\left( {\begin{array}{c}2j\\ j\end{array}}\right) ^2\left( {\begin{array}{c}3j\\ j\end{array}}\right) \left( {\begin{array}{c}p+2j\\ 3j+1\end{array}}\right) }{(-16)^j}\frac{\sigma _4}{(3j+2)(3j+4)}\\&\quad \equiv \sum _{j=0}^{\frac{p-1}{2}}\frac{\left( {\begin{array}{c}2j\\ j\end{array}}\right) ^2p(-1)^j (1+pH_{2j}-pH_j)}{(-16)^j}\frac{\sigma _4}{(3j+1)(3j+2)(3j+4)}+S_2, \end{aligned}$$

where $S_2$ is defined by the following expression with $k=(p-4)/3$,

$$\begin{aligned}&S_2=-\frac{\left( {\begin{array}{c}-\frac{1}{2}\\ k\end{array}}\right) ^2(k(1+2k)+2p(k+1)(3k+1)-2p^2(3k+1)(3k+2))}{3k+2}\\&\quad \;\times \left( \frac{\left( {\begin{array}{c}3k\\ k\end{array}}\right) \left( {\begin{array}{c}p+2k\\ 3k+1\end{array}}\right) }{3k+4}+\frac{1+pH_{2k}-pH_k}{3k+1}\right) . \end{aligned}$$

Hence, as above, by (3.3), (3.4), (3.5), Lemmas 2.5 and 2.7, we have

$$\begin{aligned}&\sum _{k=0}^{p-1}k^3\frac{D_k}{16^k}-\frac{1}{18p(p+1)}\left( {\begin{array}{c}-1/2\\ \frac{2p-2}{3}\end{array}}\right) ^2 \left( {\begin{array}{c}2p-2\\ \frac{2p-2}{3}\end{array}}\right) \left( {\begin{array}{c}p+\frac{4p-4}{3}\\ 2p-1\end{array}}\right) \\&\equiv \frac{p}{27}\sum _{j=0}^{\frac{p-1}{2}}\frac{\left( {\begin{array}{c}2j\\ j\end{array}}\right) ^2}{16^j} \left( \frac{-1}{3j+1}+\frac{-3}{3j+2}+\frac{10}{3j+4}\right) \\&\quad \;+\frac{p^2}{3}\sum _{j=0}^{\frac{p-1}{2}}\frac{\left( {\begin{array}{c}2j\\ j\end{array}}\right) ^2}{16^j} \left( \frac{1}{3j+2}+\frac{1}{3j+4}\right) -2p^3\sum _{j=0}^{\frac{p-1}{2}}\frac{\left( {\begin{array}{c}2j\\ j\end{array}}\right) ^2}{(3j+4)16^j}\\&\quad \;+\frac{p^2}{27}\sum _{j=0}^{\frac{p-1}{2}}\frac{\left( {\begin{array}{c}2j\\ j\end{array}}\right) ^2}{16^j} \left( \frac{-1}{3j+1}+\frac{-3}{3j+2}+\frac{10}{3j+4}\right) (H_{2j}-H_j)\\&\quad \;+\frac{p^3}{3}\sum _{j=0}^{\frac{p-1}{2}}\frac{\left( {\begin{array}{c}2j\\ j\end{array}}\right) ^2}{16^j} \left( \frac{1}{3j+2}+\frac{1}{3j+4}\right) (H_{2j}-H_j)+\frac{184p^2x^2}{125}\\&\equiv \left( -\frac{1}{27}+\frac{10}{27}\frac{4}{25}\right) \left( 4x^2-2p- \frac{p^2}{4x^2}\right) +\frac{1}{9}\frac{p^2}{x^2}+\frac{p}{3}\frac{4}{25}(4x^2-2p)\\&\quad \;-2p^2\frac{16x^2}{25}-\frac{10p}{27}\frac{18}{125}(4x^2-2p)+ \frac{1}{9}\frac{3}{2}\frac{p^2}{x^2}-\frac{p^2}{3}\frac{18}{125}4x^2+\frac{184p^2x^2}{125}\\&\equiv \frac{4x^2}{45}-\frac{2p}{45}+\frac{49p^2}{180x^2}\quad \ (\mathrm{{mod}}\ p^3). \end{aligned}$$

It is easy to see that

$$\begin{aligned} \left( {\begin{array}{c}2p-2\\ \frac{2p-2}{3}\end{array}}\right) \left( {\begin{array}{c}p+\frac{4p-4}{3}\\ 2p-1\end{array}}\right) \equiv -2p\quad \ (\mathrm{{mod}}\ p^2). \end{aligned}$$

In view of [7, pp. 18], we have

$$\begin{aligned} \left( {\begin{array}{c}-1/2\\ \frac{2p-2}{3}\end{array}}\right) ^2\equiv \frac{9p^2}{4x^2}\quad \ (\mathrm{{mod}}\ p^3). \end{aligned}$$

These yield

3.10 $$\begin{aligned} \sum _{k=0}^{p-1}k^3\frac{D_k}{16^k}&\equiv \frac{4x^2}{45}-\frac{2p}{45} +\frac{49p^2}{180x^2}-\frac{p^2}{4x^2}\nonumber \\&=\frac{4x^2}{45}-\frac{2p}{45}+\frac{p^2}{45x^2}\quad \ (\mathrm{{mod}}\ p^3). \end{aligned}$$

If $p\equiv 2\ (\mathrm{{mod}}\ 3)$ with $p>5$ (the case $p=5$ can be checked directly), then modulo $p^2$, we have

$$\begin{aligned}&\sum _{k=0}^{p-1}k^3\frac{D_k}{16^k}\equiv \sum _{j=0}^{\frac{p-1}{2}}\frac{\left( {\begin{array}{c}2j\\ j\end{array}}\right) ^2}{16^j}\frac{pj(1+2j)+ 2p^2(j+1)(3j+1)+p^2j(2j+1)(H_{2j}-H_j)}{(3j+1)(3j+2)(3j+4)}. \end{aligned}$$

Hence, similar to above, we have

$$\begin{aligned} \sum _{k=0}^{p-1}k^3\frac{D_k}{16^k}&\equiv \frac{p}{27}\sum _{j=0}^{\frac{p-1}{2}} \frac{\left( {\begin{array}{c}2j\\ j\end{array}}\right) ^2}{16^j}\left( \frac{-1}{3j+1}+\frac{-3}{3j+2}+\frac{10}{3j+4}\right) \\&\quad \;+\frac{p^2}{3}\sum _{j=0}^{\frac{p-1}{2}}\frac{\left( {\begin{array}{c}2j\\ j\end{array}}\right) ^2}{16^j} \left( \frac{1}{3j+2}+\frac{1}{3j+4}\right) \\&\quad \;+\frac{p^2}{27}\sum _{j=0}^{\frac{p-1}{2}}\frac{\left( {\begin{array}{c}2j\\ j\end{array}}\right) ^2}{16^j} \left( \frac{-1}{3j+1}+\frac{-3}{3j+2}+\frac{10}{3j+4}\right) (H_{2j}-H_j)\\&\equiv -\frac{p}{9}\sum _{j=0}^{\frac{p-1}{2}}\frac{\left( {\begin{array}{c}2j\\ j\end{array}}\right) ^2}{(3j+2)16^j} +\frac{p^2}{3}\sum _{j=0}^{\frac{p-1}{2}}\frac{\left( {\begin{array}{c}2j\\ j\end{array}}\right) ^2}{(3j+2)16^j}\\&\quad \;-\frac{p^2}{9}\sum _{j=0}^{\frac{p-1}{2}}\frac{\left( {\begin{array}{c}2j\\ j\end{array}}\right) ^2}{(3j+2)16^j}(H_{2j}-H_j)\\&\equiv -\frac{p}{9}\sum _{j=0}^{\frac{p-1}{2}}\frac{\left( {\begin{array}{c}2j\\ j\end{array}}\right) ^2}{(3j+2)16^j}\equiv -\frac{4}{9}R_3(p)\quad \ (\mathrm{{mod}}\ p^2). \end{aligned}$$

This, together with (3.6), (3.9) and (3.10), completes the proof of Theorem 1.1. $\square$

## Data Availability

Data sharing is not applicable to this article as no new data were created or analyzed in this study.
